# Supplementary material for: Comparative multiomics analysis of cell physiological state after culture in a basket bioreactor
Source: Sci Rep. 2022 Nov 23;12:20161. doi: 10.1038/s41598-022-24687-4 (PMC9686226; doi:10.1038/s41598-022-24687-4)
Supplement: Supplementary file 1 — Supplementary Information 1. [file 41598_2022_24687_MOESM1_ESM.zip › raw data/Proteomic raw data/2.func.anno/2.2.COG/COG.anno.pdf]

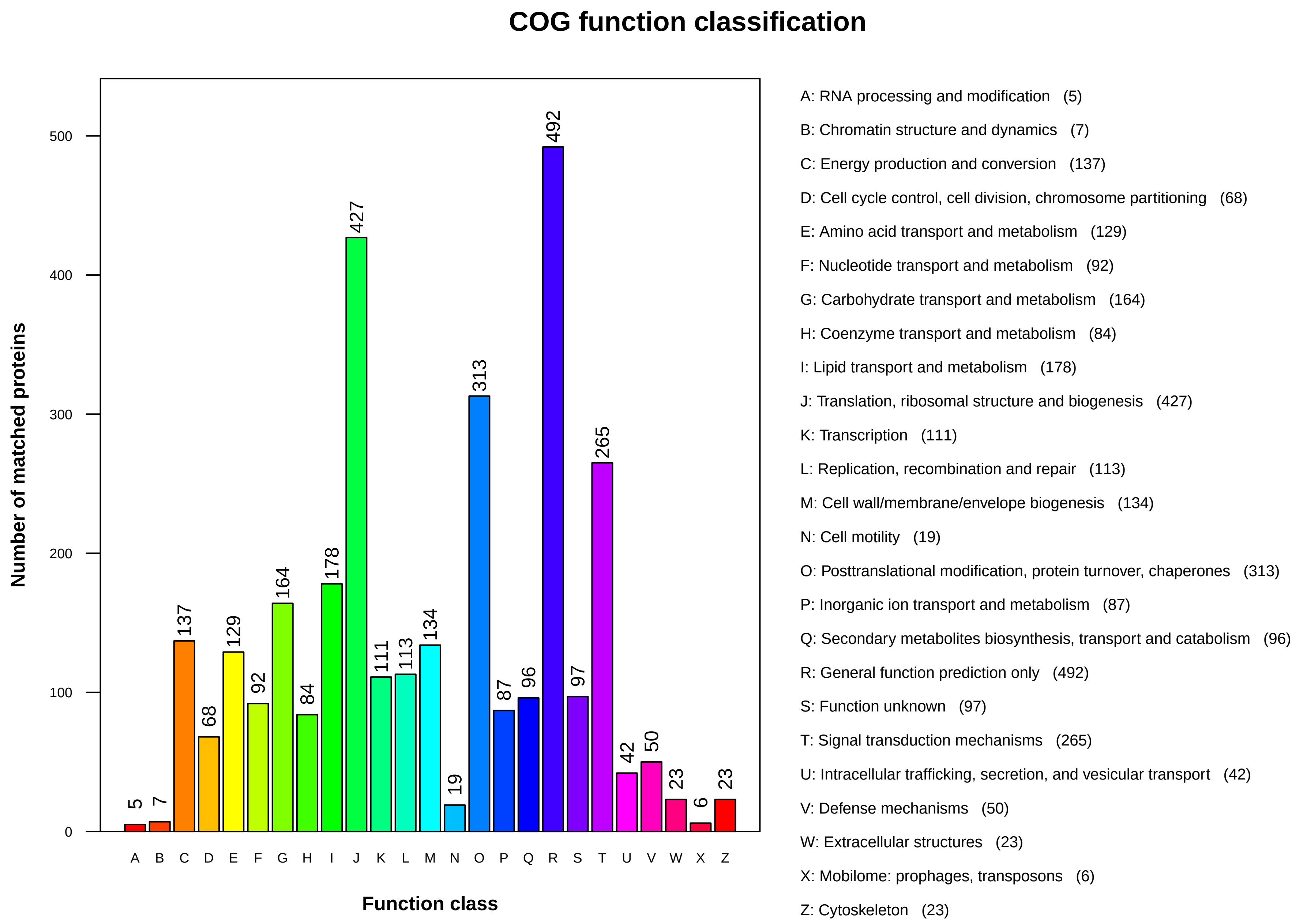
COG function classification
Number of matched proteins
0
100
200
300
400
500
5 7 137 68 129 92 164 84 178 427 111 113 134 19 313 87 96 492 97 265 42 50 23 6 23
A B C D E F G H I J K L M N O P Q R S T U V W X Z
Function class
A: RNA processing and modification (5)
B: Chromatin structure and dynamics (7)
C: Energy production and conversion (137)
D: Cell cycle control, cell division, chromosome partitioning (68)
E: Amino acid transport and metabolism (129)
F: Nucleotide transport and metabolism (92)
G: Carbohydrate transport and metabolism (164)
H: Coenzyme transport and metabolism (84)
I: Lipid transport and metabolism (178)
J: Translation, ribosomal structure and biogenesis (427)
K: Transcription (111)
L: Replication, recombination and repair (113)
M: Cell wall/membrane/envelope biogenesis (134)
N: Cell motility (19)
O: Posttranslational modification, protein turnover, chaperones (313)
P: Inorganic ion transport and metabolism (87)
Q: Secondary metabolites biosynthesis, transport and catabolism (96)
R: General function prediction only (492)
S: Function unknown (97)
T: Signal transduction mechanisms (265)
U: Intracellular trafficking, secretion, and vesicular transport (42)
V: Defense mechanisms (50)
W: Extracellular structures (23)
X: Mobilome: prophages, transposons (6)
Z: Cytoskeleton (23)
